# Detection and Analysis of Circadian Biomarkers for Metabolic Syndrome Using Wearable Data: Cross-Sectional Study

**DOI:** 10.2196/69328

**Published:** 2025-07-16

**Authors:** Jeong-Kyun Kim, Sujeong Mun, Siwoo Lee

**Affiliations:** 1 KM Data Division, Korea Institute of Oriental Medicine, 1672 Yuseong-daero, Yuseong-gu, Daejeon, Daejeon, 34054, Republic of Korea, 82 10-4654-6164, 82 42-868-9555

**Keywords:** metabolic syndrome, wearable device, digital biomarker, circadian rhythm, explainable artificial intelligence

## Abstract

**Background:**

Wearable devices are increasingly used for monitoring health and detecting digital biomarkers related to chronic diseases such as metabolic syndrome (MetS). Although circadian rhythm disturbances are known to contribute to MetS, few studies have explored wearable-derived circadian biomarkers for MetS identification.

**Objective:**

This study aimed to detect and analyze sleep and circadian rhythm biomarkers associated with MetS using step count and heart rate data from wearable devices and to identify the key biomarkers using explainable artificial intelligence (XAI).

**Methods:**

Data were analyzed from 272 participants in the Korean Medicine Daejeon Citizen Cohort, collected between 2020 and 2023, including 88 participants with MetS and 184 without any MetS diagnostic criteria. Participants wore Fitbit Versa or Inspire 2 devices for at least 5 weekdays, providing minute-level heart rate, step count, and sleep data. A total of 26 indicators were derived, including sleep markers (midsleep time and total sleep time) and circadian rhythm markers (midline estimating statistic of rhythm, amplitude, interdaily stability, and relative amplitude). In addition, a novel circadian rhythm marker, continuous wavelet circadian rhythm energy (CCE), was proposed using continuous wavelet transform of heart rate signals. Statistical tests (*t* test and the Wilcoxon rank sum test) and machine learning models—Shapley Additive Explanations, explainable boosting machine, and tabular neural network—were applied to evaluate marker significance and importance.

**Results:**

Circadian rhythm markers, especially heart rate–based indicators, showed stronger associations with MetS than sleep markers. The newly proposed CCE demonstrated the highest importance for MetS identification across all XAI models, with significantly lower values observed in the MetS group (*P*<.001). Other heart rate–based markers, including relative amplitude and low activity period, were also identified as important contributors. Although sleep markers did not reach statistical significance, some were recognized as secondary predictors in XAI-based analyses. The CCE marker maintained a high predictive value even when adjusting for age, sex, and BMI.

**Conclusions:**

This study identified CCE and relative amplitude of heart rate as key circadian rhythm biomarkers for MetS monitoring, demonstrating their high importance across multiple XAI models. In contrast, traditional sleep markers showed limited significance, suggesting that circadian rhythm analysis may offer additional insights into MetS beyond sleep-related indicators. These findings highlight the potential of wearable-based circadian biomarkers for improving MetS assessment and management.

## Introduction

Metabolic syndrome (MetS) is a multifaceted disorder that includes various metabolic conditions, such as obesity, hypertension, and diabetes. These conditions are becoming increasingly prevalent worldwide, posing significant concerns with profound implications for individual health, societal well-being, and the economy. Metabolic diseases involve complex pathophysiological mechanisms [1,2]. Promoting healthy eating habits, integrating appropriate physical activity, and effectively managing stress can significantly reduce the risk of developing MetS [1,3].

Currently, health management systems using wearable devices are gaining significant attention [4]. These systems offer personalized health care by continuously monitoring and analyzing the health status of an individual in real time. Wearable devices enable regular assessment of health-related indicators, eliminating the need for frequent hospital visits [2]. Achieving this requires the integration of various technologies, including semiconductors, artificial intelligence, and the Internet of Things [5]. Smartwatches and bands are among the most commonly used wearable devices in daily life. Smartwatches are equipped with diverse sensors such as optical heart rate (HR) monitors, electrocardiograms, and inertial sensors, enabling the collection of vital signs such as step count, HR, blood pressure, pulse, and oxygen saturation [5].

Wearable devices can continuously monitor an individual’s vital signs in real time. This involves the continuous tracking of important indicators related to MetS, such as HR, blood pressure, and activity levels, aiding in the real-time assessment of an individual’s health status and the early detection of anomalies [2,6]. The daily data generated by wearable devices can help identify factors influencing the onset and progression of MetS. Recently, there has been a significant increase in attention to the daily monitoring of sleep and circadian rhythms using wearable devices [7,8]. Circadian rhythms are biochemical, physiological, or behavioral processes that occur in living organisms in approximately 24-hour cycles. Circadian rhythm markers are indicators to assess or measure the circadian rhythm state of a living organism [8,9]. Sleep and circadian rhythm biomarkers derived from activity-based wearable devices are now applied in studies on various conditions, including obesity [7], depression [8,9], cognitive function [10], prematurity [11], and mortality [12]. Common sleep markers include sleep timing (sleep onset and sleep offset), sleep duration (total sleep time), midsleep time, and wake after sleep onset [7,8]. Circadian rhythm markers are assessed using both parametric methods, such as amplitude, acrophase, midline estimating statistic of rhythm (MESOR), and circadian quotient (CQ) via cosinor analysis [8,12], and nonparametric methods, such as interdaily stability (IS), intradaily variability (IV), and relative amplitude (RA) [9-11]. Given the periodic nature of circadian rhythms, energy-based methods have been proposed. For example, singular value decomposition (SVD) has been used to calculate energy values from activity data, suggesting potential markers for cognitive function [10]. Traditional methods for measuring signal energy, such as Fourier transform, discrete cosine transform, and wavelet analysis, have been used in various biosignal analysis studies [13]. In this study, we propose a novel marker called continuous wavelet circadian rhythm energy (CCE) using continuous wavelet transform (CWT) for time-frequency analysis of HR as a circadian rhythm marker.

Previously, circadian rhythm analysis using wrist-worn wearable devices focused on activity-centered markers through actigraphy [9,10,12]. However, recently, the demand for circadian rhythm analysis based on HR and step counts provided by smartwatches and bands has been increasing [7,8]. Wearable devices often underestimate and overestimate the number of steps in free-living environments. Therefore, circadian rhythm markers in this study were obtained by integrating 5 days of continuous data, which addressed inaccuracies in step count measurements by reflecting the overall trends in sleep and activity. In addition, this study can compensate for incorrectly measured step counts by exploring HR-based markers rather than step counts.

The majority of the previous studies detected everyday features obtained from wearable devices and analyzed the features limited to statistical techniques without incorporating artificial intelligence technology [9-12]. Explainable artificial intelligence (XAI) techniques that interpret predictive results are gaining attention in the analysis of biosignals and health care information [5,7]. Specifically, research on circadian rhythm analysis based on wearable devices in relation to MetS is still in its early stages. This area requires multifaceted analysis, including the development of circadian rhythm biomarkers specifically tailored for MetS and the application of XAI for data interpretation.

Therefore, in this study, we detected circadian rhythm markers based on step count and HR to develop a wearable-based circadian rhythm marker for MetS and analyzed circadian rhythm markers important for MetS through artificial intelligence technologies such as Shapley Additive Explanations (SHAP), explainable boosting machine (EBM), and Tabular neural Network models (TabNet). Integration with XAI in marker analysis can make the results more actionable in health care applications due to its transparency and interpretability.

## Methods

### Recruitment

The Korean Medicine Daejeon Citizen Cohort (KDCC) dataset was used to validate the proposed biomarker. The data from the KDCC, spanning from 2020 to 2023, encompassed demographic, lifestyle, clinical, and biochemical measurements collected from 2000 participants. Anthropometric data, including height, weight, BMI, waist circumference, and hip circumference, were measured under standardized conditions. Height and weight were recorded using a digital stadiometer (BSM370, InBody), and BMI was calculated as weight (kg) divided by the square of height (m²). Waist circumference was measured at the level of the navel, and hip circumference was measured at the widest part of the hips using a measuring tape (Hoechstmass-Rollvix, Germany).

Systolic blood pressure, diastolic blood pressure, and resting HR were measured using an automatic blood pressure cuff (FT-500R PLUS, Jawon Medical, South Korea). Measurements were taken after at least 5 minutes of rest, with an additional 1-minute interval between repeated readings. The average of 2 measurements was used for analysis. Venous blood samples (22.5 mL) were collected in the morning after overnight fasting. After 30 minutes, blood samples were centrifuged at 3450 rpm for 10 minutes, and all samples were transported within 24 hours to Seoul Clinical Laboratories (Seoul, Korea) for analysis. The biochemical analysis included complete blood count, kidney and liver function tests, lipid profile (triglycerides, high-density lipoprotein, low-density lipoprotein, and total cholesterol), glucose metabolism markers, thyroid function tests, and inflammation markers [14].

Daily data was obtained using Fitbit Versa or Inspire 2 wristbands (Fitbit) [15]. Of the initial 2000 participants enrolled in the KDCC cohort, not all consented to wearable device use. Consequently, 745 participants provided usable Fitbit data. Participants who wore Fitbit for less than 5 consecutive weekdays or had data for more than 6 hours of nonwearing in a 24-hour period were excluded from the 745 participants. Finally, 500 of the 745 participants were included in the analysis.

MetS was defined as meeting 3 or more of the following criteria, based on a 5-criteria system that uses the criteria described in the modified third national cholesterol education program adult treatment panel by modifying the waist circumference cutoff point for Koreans [15] in Textbox 1.

Textbox 1. **Third National Cholesterol Education Program Adult Treatment Panel.**

- Waist circumference (≥90 cm for men, ≥85 cm for women)
- Systolic blood pressure (≥130 mm Hg) or diastolic blood pressure (≥85 mm Hg), or current use of antihypertensive medications
- Low high-density lipoprotein cholesterol levels (<40 mg/dL in men and <50 mg/dL in women) or use of medication for lipid abnormalities
- Triglyceride level (≥150 mg/dL) or medication for lipid abnormalities
- Fasting blood glucose level (≥100 mg/dL) or medication for type 2 diabetes

Among 500 participants (158 males and 342 females), 88 participants (44 males and 44 females) met the criteria for MetS, and 184 participants (28 males and 156 females) did not meet any of the 5 criteria and were classified as non-MetS.

### Ethical Considerations

This study was conducted following the Declaration of Helsinki and was approved by the institutional review board of Dunsan Korean Medicine Hospital, Daejeon University (approval number DJDSKH-17-BM-12) and the Korea Institute of Oriental Medicine (approval number I-1703/002‐002). Before participation, all individuals provided written informed consent, including consent for collecting human biological materials. Participants were informed that they could withdraw their consent at any time without any consequences.

Participants received US $36 as compensation for transportation costs. Written consent was obtained from the individuals involved for the publication of potentially identifiable images or data. All included images were reviewed to ensure that personal identities could not be inferred. This study was designed and conducted in compliance with ethical standards for human research, ensuring participant rights, privacy, and safety throughout the study process.

### Circadian Rhythm and Sleep Indicator

Wearable data included minute-level HR and step-count records collected from Fitbit devices. In addition, the sleep duration records provided by Fitbit were collected. Notably, the minute-level HR records from the Fitbit devices occasionally had missing values, represented by zeros. Missing values were converted to the nearest HR. Missing data mainly consisted of long periods of nonwearing, such as during charging or showering. Spline interpolation resulted in exaggerated patterns, such as sudden increases or decreases in HR during long periods of nonwearing. Given these considerations, linear interpolation was chosen to minimize distortion and better handle long intervals.

The most commonly used indicators in sleep analysis are midsleep time (MST), total sleep time (TST), and wake after sleep onset [7,8]. MST is used to determine phenotype, such as whether an individual is a morning or evening sleeper. Knowing MST and TST can reveal the amount of sleep and phenotype, but it does not provide information about sleep efficiency. Therefore, the ratio of total sleep time to total time spent in bed for sleep is used. However, it is difficult to estimate sleep efficiency with step count and HR data provided by Fitbit.

Cosinor analysis is frequently used to analyze circadian rhythms based on activity and HR. Cosinor analysis fits sine waves to time series using the least squares method [16]. It is often used in biological time series analysis and can be applied to time series with nonuniform intervals. Cosinor analysis expresses observed time series data as a cosine function, as shown in Equation 1. Y(t) is the observation at time t, M is the mean (MESOR), A is the amplitude, τ is the period, t is the time, and φ is the phase. MESOR is the middle value of periodic oscillations and represents the central tendency of the data. The amplitude is the amount of variation in the period and represents how much the data deviates from the mean. The phase is the location of the variation corresponding to a specific time in the period and can mean the time when the peak of the data occurs. Amplitude, acrophase, MESOR, and CQ (amplitude or MESOR) are used as cosinor-related indicators [8,9,12].


(1) $$Y\left(t\right)=M+A\cos\left(\frac{2\pi t}{\tau }+\varphi \right)$$

Nonparametric indicators used in circadian rhythm studies related to daily activity include RA, IV, and IS, which evaluate the stability and variability of individual activity patterns and circadian rhythms [8-10]. RA is an index that evaluates the amplitude of activity in circadian rhythms and is calculated by the difference in activity between the maximum and minimum activity times as shown in Equation 2. M10 means the mean activity during the 10 hours with the highest activity during the day, and L5 means the mean activity during the 5 hours with the lowest activity during the day. The higher the RA, the greater the difference in activity between day and night, indicating a more distinct circadian rhythm.

IV is an index that evaluates the variability of activity patterns within a day, as shown in Equation 3. N is the total number of measured time data, $Y_{t}$ is the activity at time t, and $\overset{-}{Y}$ is the mean activity over all times. The numerator of Equation 3 is the sum of the squares of the activity variance between consecutive time periods. The larger the change in activity between time periods during the day, the larger the IV value. The denominator is the total change in activity by time, which expresses the variability for the mean activity during the day.

IS is an index that evaluates how consistently activity levels are maintained at the same time across multiple days. It indicates how regular and consistent the activity patterns are maintained by the time zone of the day. In Equation 4, N is the total number of measured time data, S is the number of data per day, and ${\overset{-}{x}}_{h}$is the hourly mean. The numerator represents the squared deviations of the hourly means from the overall mean, whereas the denominator reflects the total variance of the entire series. As the ratio of these 2 terms increases, the IS value becomes larger, and activity patterns that recur at identical clock times on successive days yield higher IS values.


(2) $$RA=\frac{M10-L5}{M10+L5}$$

(3) $$IV=\frac{N\sum_{t=2}^{N}{\left(Y_{t}-Y_{t-1}\right)}^{2}}{(N-1)\sum_{t=1}^{N}{\left(Y_{t}-\overset{-}{Y}\right)}^{2}}$$

(4) $$IS=\frac{N\sum_{h=1}^{S}{\left({\overset{-}{x}}_{h}-\overset{-}{x}\right)}^{2}}{S\sum_{i=1}^{N}{\left(x_{i}-\overset{-}{x}\right)}^{2}}$$

Dimension reduction technology is being utilized as a method of analyzing biosignals, which is a method of converting high-dimensional data into low-dimensional data to easily visualize or analyze the data [17,18]. Dimension reduction is mainly used to reduce computational costs, remove noise, and express data more concisely while preserving the features of the data. Principal component analysis, linear discriminant analysis, and SVD are used as dimension reduction methods. In a study that applied dimension reduction technology to circadian rhythm, circadian activity rhythm energy (CARE) was proposed to detect the energy of subsignals with a cycle of less than 24 hours through singular spectrum analysis based on SVD to analyze the circadian rhythm of cognitive function. CARE showed a higher correlation coefficient than RA for melatonin amplitude [10].

The activity and HR obtained from wearable devices have a 24-h periodicity. Wavelet transform (WT) is used as a method to analyze signals that have time periodicity [19]. WT is a powerful tool that can analyze signals in the time and frequency domains simultaneously. WT analyzes frequency information in each time interval by decomposing them into wavelets of various sizes. CWT calculates wavelet changes for all times. Morlet wavelet is a sinusoidal waveform that is attenuated by a Gaussian curve, which is very advantageous in detecting the temporal changes of specific frequency components, allows for high-resolution frequency analysis, and captures continuous and periodic signal components well [14,20]. The CWT for a signal x(t) is obtained by computing the inner product with the Morlet wavelet. In Equations5  6, $W_{x}\left(a,b\right)$ is the CWT coefficient of a given signal x(t), a is a scale factor that controls the frequency variation. A smaller a corresponds to a higher frequency component, and a larger a corresponds to a lower frequency component. b is a shift parameter that controls the variation in time, and the signal is analyzed along the time axis as b changes. $\psi^{*}$is the complex combination of the Morlet wavelet, t is time, and x(t) represents the signal to be analyzed. $\psi \left(t\right)$ is the Morlet wavelet function, $\omega_{0}$ is the center frequency, and i is the imaginary unit. The magnitude (energy) of the CWT coefficients $W_{x}\left(a,b\right)$ represents how strongly the signal x(t) exists at a given scale a and time b. This energy is used to generate a spectrogram, which is a map that shows the intensity of the signal in the time-frequency plane. The center frequency means that the Morlet wavelet is optimized for analyzing signals in a specific frequency band. To obtain circadian rhythm markers for the acquired 5-day HR and step count per min, spectrograms were obtained for each signal, and then the total energy of the center frequency was derived as a marker by adding up all the energy over time for each center frequency. The proposed CWT-based circadian rhythm energy is called CCE.


(5) $$\psi (t)=\pi^{-\frac{1}{4}}∙e^{i\omega_{0}t}∙e^{-\frac{t^{2}}{2}}$$

(6) $$W_{x}(a,b)=\frac{1}{\sqrt{a}}\int_{-\infty }^{\infty }x(t)∙\psi^{*}(\frac{t-b}{a})dt$$

### Feature Selection and XAI

XAI is a methodology used to interpret the results of machine learning models. XAI enabled us to analyze the contribution of the features to the model outcomes. The local interpretable model-agnostic explanation (LIME) and SHAP are commonly used XAI algorithms, particularly for analyzing tabular data. As LIME often focuses on local explanations, it may not effectively explain the global behavior of the models. SHAP is a model-agnostic post hoc algorithm that is gaining attention. SHAP offers special implementations of tree-based models and provides more accurate and efficient explanations [21]. A tree-based model was used to identify MetS. Thus, SHAP was used to analyze the important features. SHAP provides insight into the contribution of each feature to the model outcomes, considering both the positive and negative impacts of wearable-based daily life features on the identification of MetS. XGBoost was used as the SHAP learning model, and the resulting SHAP values were as follows [5]:


(7) $$\emptyset_{i}(v)=\sum_{S\in N\left\{i\right\}}\frac{|S|!(n-|S|-1)!}{n!}(v(S\cup \left\{i\right\}-v(S))$$

XGBoost is a boosting model for decision trees that enhances the performance of gradient-boosting machines in terms of their speed. Boosting models iteratively update the parameters of previous classifiers to create a more powerful classifier, thereby increasing the accuracy and reducing the gradient of the loss function [5].

EBM is an interpretable structure of the model itself, unlike SHAP, which explains how each feature contributes to the prediction [22]. EBM is based on a generalized additive model (GAM), and GAM models the relationship between features and target variables as a sum of functions for each feature [23]. EBM can visualize how each feature contributes to the prediction so that users can easily understand the influence of individual features on the results. Equation 8 represents GAM, where $\beta_{0}$ is a constant term, and $f_{i}\left(x_{i}\right)$ is a function learned for each feature, indicating how each feature contributes to the prediction. g is a link function that adjusts GAM to various settings, such as regression or classification. EBM learns each function $f_{i}\left(x_{i}\right)$ of GAM using boosting techniques. Boosting is a method of sequentially learning multiple weak models and correcting errors made by previous models, as shown in equation 9. $h_{m}\left(x_{i}\right)$ is the m-th weak model, $\alpha_{m}$ is the weight of the m-th weak model, and M represents the boosting step. EBM, including GAM and boosting techniques, is shown in Equation 10, and EBM can automatically detect pairwise interactions between each feature that are important for prediction and include them in the model, as shown in Equation 11.


(8) $$g(E[y])=\beta_{0}+f_{1}(x_{1})+f_{2}(x_{2})+f_{3}(x_{3})+\cdots +f_{n}(x_{n})$$

(9) $$f_{i}\left(x_{i}\right)=\sum_{m=1}^{M}\alpha_{m}h_{m}\left(x_{i}\right)$$

(10) $$g\left(E[y]\right)=\beta_{0}+\sum_{i=1}^{n}\sum_{m=1}^{M}\alpha_{m}h_{m}(x_{i})$$

(11) $$g\left(E[y]\right)=\beta_{0}+\sum f_{i}(x_{i})+\sum f_{ij}(x_{i},x_{j})$$

In addition to EBM and XGBoost, TabNet, a deep learning architecture designed for tabular data, was used. TabNet leverages a novel attention mechanism that automatically selects relevant features and effectively learns their interactions [24]. TabNet is particularly well-suited for analyzing complex datasets because it handles missing values and displays the most influential features for prediction, providing interpretability. In TabNet, the method to automatically evaluate the importance of each feature to the model is related to the attention mechanism and Gated Linear Units (GLU). To evaluate the importance of each feature in a given input X, the attention weights $W_{a}$ are calculated as in Equation 12. Z is the input value of the current layer, A is the learnable weight, and P is the prior-scale term that reflects how frequently the corresponding feature has been selected in earlier steps. Consequently, A becomes a probability vector indicating the importance of each feature. TabNet performs prediction through features selected from each layer, and the set of selected features is defined as in Equation 13. GLU is used in the process of performing prediction using selected features. GLU can emphasize or suppress input features through nonlinear transformation, as shown in Equation 14. Wis a weight matrix, b is a bias, and *σ* is a sigmoid activation function, which provides a ratio representing the importance of each feature. The output computed through the attention weights and GLU of each layer evaluates the importance of each feature to the final prediction.


(12) $$A=Sparsemax\left(P\cdot (W_{a}Z)\right)$$

(13) $$X_{selected}=A∙X$$

(14) $$GLU(x)=(W_{1}x+b_{1})\cdot \sigma (W_{2}x+b_{2})$$

The XGBoost parameters used were booster=gbtree and objective=binary logistic. The other parameters were set to the default values in Python (v3.10.9), NumPy (v1.23.5), and scikit-learn (v1.2.2). EBM was implemented using the interpret library version 0.6.4, and TabNet was implemented using pytorch-tabnet version 4.1.0 with max_epochs=2000 and batch_size=32.

## Results

### Associations of Sleep and Circadian Rhythm Indicators With MetS

Based on HR, step count, and sleep data obtained from wearable devices, bio-digital markers of circadian rhythm for MetS were detected and the significance of the markers was analyzed. For the analysis, continuous wearable data for 5 days during the week of 88 MetS and 184 non-MetS obtained from smart bands on the wrist were used. Demographic and clinical information used to determine MetS are shown in Table 1. The age of the MetS group was 48.94 years, which was 4 years older than that of the non-MetS group. The average BMI of MetS was 27.9. Systolic blood pressure, diastolic blood pressure, triglyceride, high-density lipoprotein, and glucose used to determine MetS all showed statistically significant results (*P*<.001). The *P* value was obtained using an independent *t* test.

**Table 1.** Demographic and clinical characteristics of MetS and non-MetS groups. Demographic data (age and sex) and clinical variables, including BMI, waist circumference, blood pressure, lipid profile, and glucose levels, are presented for participants classified into MetS (n=88) and non-MetS (n=184) groups. Statistical significance was tested using *t* tests.

	Non-MetS^a^	MetS	*P* value
Sex, n (%)			
Male	28 (38.9)	44 (61.1)	
Female	156 (78.0)	44 (22.0)	
Age (years), mean (SD)	44.04 (6.48)	48.94 (6.92)	<.001
BMI (kg/m^2^), mean (SD)	21.70 (2.08)	27.90 (2.94)	<.001
Waist (cm), mean (SD)			
Male	81.82 (5.28)	96.98 (7.41)	<.001
Female	75.88 (5.11)	90.45 (8.04)	<.001
SBP^b^ (mm Hg)	110.32 (8.98)	130.68 (15.42)	<.001
DBP^c^ (mm Hg)	67.97 (8.80)	83.07 (11.31)	<.001
Triglyceride (mg/dL)	78.29 (26.62)	210.01 (102.54)	<.001
HDL^d^ (mg/dL)	68.17 (14.21)	46.40 (11.35)	<.001
LDL^e^ (mg/dL)	123.02 (33.19)	124.13 (35.95)	.74
Total_Cholesterol (mg/dL)	204.94 (35.05)	200.51 (42.11)	.45
Glucose (mg/dL)	82.97 (6.52)	99.89 (20.84)	<.001

a MetS: metabolic syndrome.

b SBP: systolic blood pressure.

c DBP: diastolic blood pressure.

d HDL: high-density lipoprotein.

e LDL: low-density lipoprotein.

Twenty-six indicators were identified as sleep and circadian rhythm markers. The sleep-related indicators consist of 4 measures: the mean and SD of MST and TST. MESOR, amplitude, acrophase, and CQ for both step count and HR, derived from cosinor analysis, make up 8 indicators. In total, 10 indicators, including L5, M10, RA, IS, and IV for step count and HR, were calculated as nonparametric indicators. In addition, one indicator was based on dimension reduction proposed in a previous study, and 3 indicators using CCE based on HR were introduced in this study.

**Figure 1.**
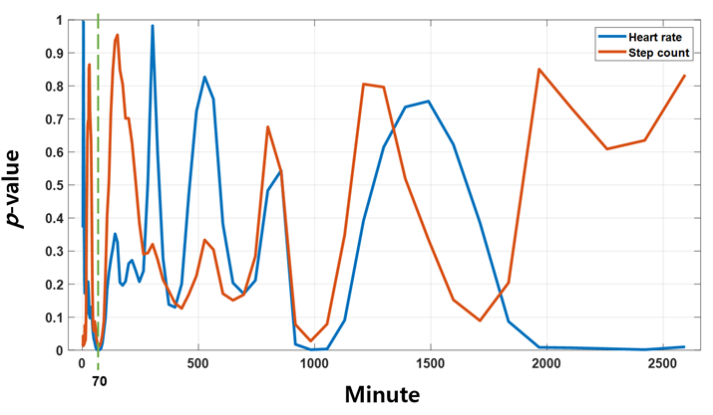
Statistical significance of continuous wavelet transform energy derived from step count and heart rate (HR) data across different central frequencies in the identification of metabolic syndrome (MetS). *P* values for each frequency range compare MetS (n=88) and non-MetS (n=184) groups, using HR and step count data recorded from Fitbit devices over 5 weekdays. HR circadian rhythm energy (CCE) showed statistical significance (*P*<.001) at 70 min and 1000 min, whereas step count CCE did not reach this threshold.

**Figure 2.**
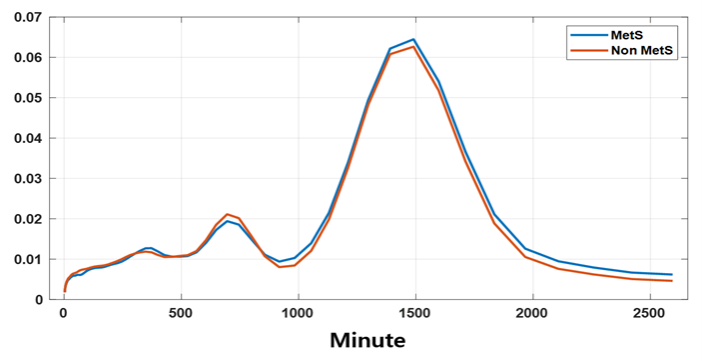
Comparison of heart rate (HR) energy at different central frequencies between metabolic syndrome (MetS) and non-MetS groups based on continuous wavelet transform analysis of wearable data. HR energy peaked at 1440 min (circadian cycle) and 700 min. In the 69‐80 min range, MetS energy was lower than non-MetS, while in the 900‐1100 min range, MetS energy was higher.

The statistical significance of MetS in the results obtained by analyzing the energy of the CWT based on the central frequency of the circadian rhythm for step count and HR, in order to detect MetS markers related to the CCE proposed in this study, is shown in Figure 1. The CCE value for step count showed the lowest *P* value at a central frequency of 70 minutes, but it did not reach a value below .001. However, HR exhibited statistical significance below 0.001 at central frequencies of 70 minutes and 1000 minutes. Therefore, the total CCE value was obtained for the central frequencies between 69 and 80 minutes (midfrequency) and between 900 and 1100 minutes (low-frequency). In addition, the ratio of the 2 central frequency ranges (mid_low ratio) was calculated as a single indicator. The energy of HR according to the central frequency reaches its highest at the 1440-minutes circadian cycle and also shows high energy in the 700-minutes range (Figure 2). In the central frequency range of 69‐80 minutes, the energy of MetS is lower than that of non-MetS, while in the 900‐1100-min range, the energy of MetS is higher.

In Tables2  3, the means, SD, and statistical significance of 26 indicators for MetS and non-MetS can be observed. Statistical significance was assessed using parametric testing (independent *t* test) and nonparametric testing (Wilcoxon rank sum test) [25].

**Table 2.** Comparison of sleep, cosinor, and nonparametric circadian rhythm indicators between metabolic syndrome (MetS) and non-MetS groups in Korean adults. Means, SD, and statistical tests (*t* test and Wilcoxon rank-sum test) are reported for each indicator derived from wearable-recorded step count and heart rate data, including sleep duration, midline estimating statistic of rhythm, amplitude, relative amplitude, interdaily stability, and others.

Indicator		Non-MetS	MetS	*t* test (*P* value)	Wilcoxon (*P* value)
Sleep					
Midsleep time	Mean	207.55 (36.36)	207.08 (28.06)	.92	.66
	SD	39.76 (24.48)	45.00 (35.50)	.16	.19
Total sleep time	Mean	414.92 (72.72)	410.70 (57.33)	.63	.46
	SD	79.50 (48.96)	84.00 (44.81)	.47	.25
Cosinor					
Midline estimating statistic of rhythm	SC^a^	7.37 (3.63)	6.42 (2.44)	.027	.102
	HR^b^	71.89 (6.39)	75.05 (7.23)	<.001	<.001
Amplitude	SC	6.61 (3.76)	5.65 (2.46)	.030	.102
	HR	11.66 (3.99)	11.22 (3.29)	.37	.47
Acrophase	SC	14.77 (2.06)	14.32 (1.83)	.084	.11
	HR	14.97 (1.95)	15.00 (1.89)	.899	.75
Circadian quotient	SC	0.89 (0.21)	0.89 (0.20)	.818	.79
	HR	0.16 (0.05)	0.15 (0.05)	.076	.04
Nonparametric					
L5	SC	0.172 (0.307)	0.203 (0.239)	.41	<.001
	HR	61.60 (6.43)	65.96 (7.25)	<.001	<.001
M10	SC	12.80 (6.42)	11.02 (4.39)	.02	.07
	HR	83.51 (7.24)	86.18 (7.68)	.006	.01
Relative amplitude	SC	0.973 (0.043)	0.961 (0.043)	.03	<.001
	HR	0.152 (0.04)	0.134 (0.03)	<.001	<.001
Interdaily stability	SC	0.507 (0.165)	0.440 (0.129)	<.001	<.001
	HR	0.684 (0.142)	0.630 (0.130)	.003	.002
Intradaily variability	SC	1.246 (0.311)	1.249 (0.274)	.93	.91
	HR	0.535 (0.157)	0.495 (0.156)	.05	.04

a SC: step count.

b HR: heart rate.

**Table 3.** Comparison of dimension reduction and frequency analysis values in metabolic syndrome (MetS) and non-MetS groups. Mean values, SD, and statistical significance for frequency-based circadian rhythm markers and dimension reduction indicators are presented. Statistical significance was assessed using independent *t* tests and Wilcoxon rank sum tests.

Indicator		Non-MetS	MetS	- test (*P* value)	Wilcoxon (*P* value)
SSA_CARE	SC^a^	0.066 (0.040)	0.061 (0.030)	.37	.69
CCE^b^					
Mid-Frequency	HR^c^	0.029 (0.010)	0.024 (0.008)	<.001	<.001
Low-Frequency	HR	0.029 (0.013)	0.034 (0.015)	.003	.003
Mid_Low Ratio	HR	1.247 (0.859)	0.865 (0.466)	<.001	<.001

a SC: step count.

b CCE: circadian rhythm energy.

c HR: heart rate.

Sleep-related indicators did not show statistical significance for MetS (*P*<.05). For the Cosinor and non-parametric methods, step count showed statistical significance with Amplitude (MetS was 0.96 smaller, *P*=.03), L5 (MetS was 0.031 larger, *P*<.001), RA (MetS was 0.012 smaller, *P*<.001), and IS (MetS was 0.067 smaller, *P*<.001). HR showed statistical significance with MESOR (MetS increased by 3.14, *P*<.001), L5 (MetS increased by 4.36, *P*<.001), M10 (MetS increased by 2.67, *P*=.012), RA (MetS decreased by 0.018, *P*<.001), IS (MetS decreased by 0.054, *P*=.002), and IV (MetS decreased by 0.04, *P*=.04). The CARE method based on step count did not show statistical significance for MetS. In this study, the proposed indicators CCE showed statistical significance in midfrequency (MetS decreased by 0.005, *P*<.001), low-frequency (MetS increased by 0.005, *P*=.003), and mid_low ratio (MetS decreased by 0.382, *P*<.001).

### XAI-Based Importance Analysis of Sleep and Circadian Rhythm Indicators for MetS

**Figure 3.**
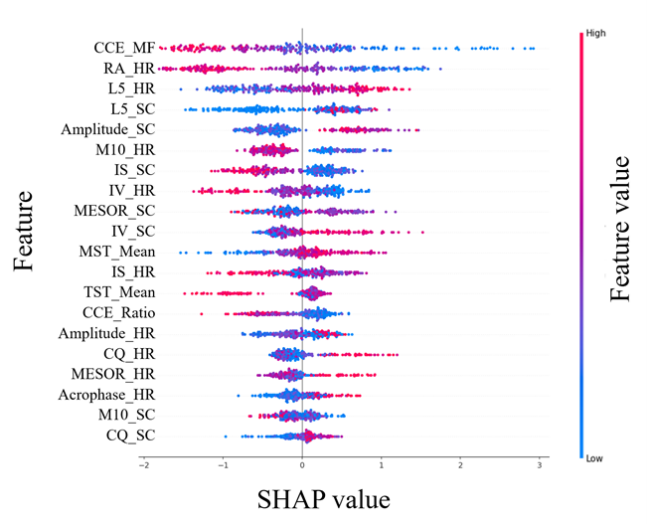
Important indicators identified for metabolic syndrome (MetS) using Shapley Additive Explanations (SHAP). Importance analysis was conducted on 26 sleep and circadian rhythm indicators using SHAP with an extreme gradient boosting (XGBoost) model. The color gradient from red to blue represents the values of each indicator, with higher values in red. The x-axis indicates the contribution direction, where positive values correspond to a higher contribution to predicting MetS, while negative values indicate a higher contribution to non-MetS.

An importance analysis was conducted on the 26 detected sleep and circadian rhythm indicators using SHAP (with XGBoost), EBM, and TabNet. Figure 3 presents the SHAP plot, where the color gradient from red to blue reflects the value of each indicator, with higher values appearing in red. The x-axis represents the contribution direction: positive values indicate a higher contribution to predicting MetS, while negative values correspond to a higher contribution to non-MetS. Indicators positioned higher on the y-axis are considered more important.

**Figure 4.**
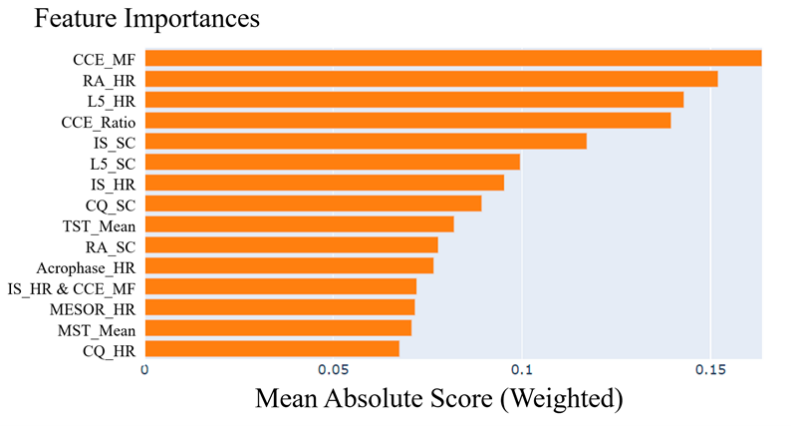
Important indicators identified for metabolic syndrome (MetS) using explainable boosting machine (EBM). Importance values of 26 sleep and circadian rhythm indicators were analyzed using EBM. The midfrequency of continuous wavelet circadian energy (CCE_MF) exhibited the highest importance for distinguishing MetS.

Figures4  5 display the importance values of indicators using EBM and TabNet, respectively. Among the three models, the midfrequency of CCE (CCE_MF), proposed in this study, exhibited the highest importance for identifying MetS-related circadian rhythm patterns. In addition, relative amplitude of HR (RA_HR) consistently ranked among the top 3 indicators across all three methods. These findings highlight the critical role of both CCE_MF and RA_HR in distinguishing between MetS and non-MetS groups.

**Figure 5.**
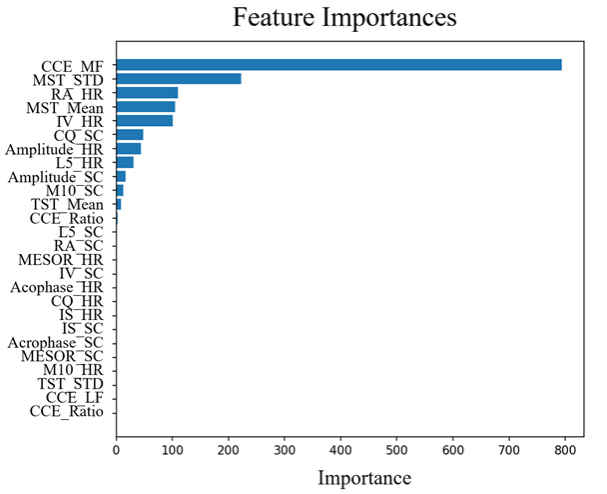
Important indicators identified for metabolic syndrome (MetS) using Tabular Neural Network. Feature importance was analyzed using TabNet, a deep learning model optimized for tabular data. Similar to explainable boosting machine, CCE_MF showed high importance in distinguishing MetS, while RA_HR also remained among the top-ranked indicators.

**Figure 6.**
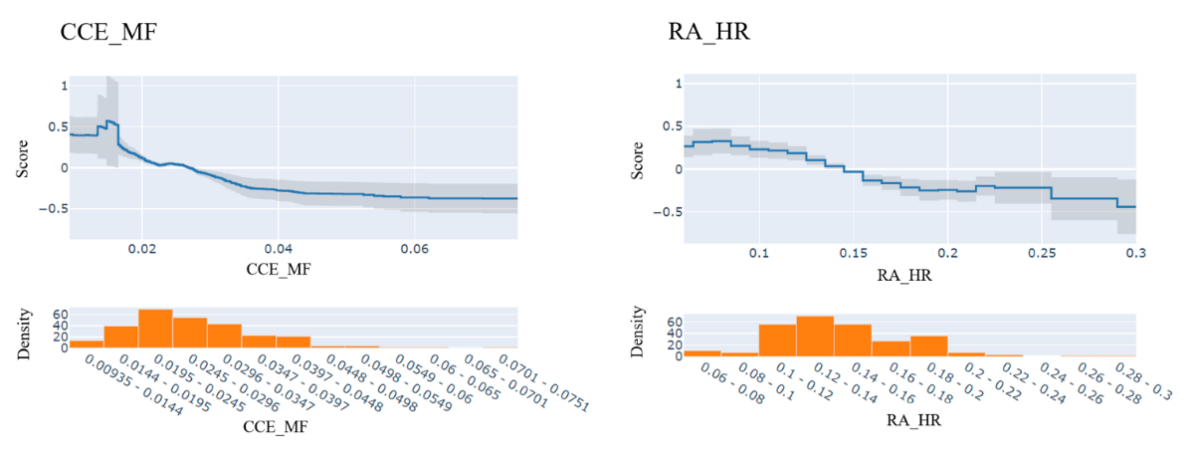
Score changes for CCE_MF and RA_HR identified by explainable boosting machine in metabolic syndrome (MetS) classification. Lower CCE_MF values were associated with a higher MetS risk, with most MetS cases clustered between 0.0195‐0.0245, while non-MetS values ranged from 0.03‐0.08. For RA_HR, values above 0.15 increased the likelihood of non-MetS, while those exceeding 0.25 were linked to a reduced probability of MetS. CCE: circadian rhythm energy; CCE_MF: midfrequency of CCE; RA_HR: relative amplitude of heart rate.

Apart from CCE_MF and RA_HR, differences in importance rankings were observed between the tree-based models (SHAP and EBM) and the deep learning-based TabNet. In the SHAP plot, both CCE_MF and RA_HR showed a higher contribution to MetS as their values decreased. Figure 6 further visualizes the changes in scores based on CCE_MF and RA_HR using EBM. The average value of CCE_MF was 0.024 for the MetS group and 0.029 for the non-MetS group, with most data points concentrated in the range of 0.0195‐0.0245. A lower CCE_MF value was associated with a higher contribution to MetS, while non-MetS data were distributed over a broader range from 0.03 to 0.08.

Similarly, RA_HR values were distributed between 0.1 and 0.2, with a shift observed in the EBM plot. When RA_HR exceeded 0.15, its contribution to non-MetS increased, while values above 0.25 were associated with a reduction in the probability of MetS.

Among the top 10 indicators, SHAP identified 5 HR-related and 5 SC-related indicators. EBM included 5 HR-related, 4 SC-related, and 1 sleep-related indicator, while TabNet identified 5 HR-related, 3 SC-related, and 2 sleep-related indicators. Across all three methods, HR-related indicators showed a high contribution to MetS. Notably, CCE_MF, RA_HR, and L5_HR consistently appeared as key contributors across all models.

The results presented above are based on the analysis of the entire dataset. To evaluate the robustness of the marker and model, the data were cross-validated 5 times. The proposed CCE_MF marker showed the highest importance in 3 of the 5 results for SHAP and was explained as the second most important marker, after RA_HR, in two results. In the EBM model, CCE_MF showed the highest importance in all five times. In the TabNet model, CCE_MF was selected within the top 10 markers, but the most important marker varied, so the probability was not shown. Therefore, the reliability of the results of the TabNet model was low.

Additionally, an analysis was conducted using SHAP, EBM, and TabNet to evaluate the impact of key demographic variables, including age, sex, and BMI, on the importance of sleep and circadian rhythm variables in the MetS prediction model.

As presented in Table 1, age, sex, and BMI are closely associated with MetS and may influence the assessment of the independent contributions of other variables when included in the model. Therefore, by comparing models that include and exclude these variables, the independent importance of sleep and circadian rhythm markers can be more clearly analyzed. The top 10 indicators in SHAP, EBM, and TabNet, when age, sex, and BMI were included, are as follows: (1) SHAP: BMI, Age, IS_SC, CCE_MF, MST_Mean, Amplitude_HR, RA_HR, M10_HR, M10_SC, and Amplitude_SC; (2) EBM: BMI, Age, Sex, CCE_MF, IS_SC, RA_HR, Age & BMI, CCE_Ratio, CQ_SC and BMI, and L5_HR; and (3) TabNet: BMI, TST_Mean, Sex, L5_SC, CCE_MF, MST_Mean, Age, IS_SC, L5_HR, and Amplitude_HR.

In models that included age, sex, and BMI, these variables significantly impacted prediction, leading to a relative decrease in the importance of some sleep and circadian rhythm markers. However, when age, sex, and BMI were excluded, the proposed CCE_MF consistently ranked among the top 3 most important markers across all methods and was identified as the most significant marker. The results indicate that the CCE_MF marker exerts a strong independent influence on MetS prediction even after accounting for potential confounding factors.

Notably, IS_SC showed a higher contribution than RA_HR and L5_HR, which is a finding worth highlighting. The findings confirm that CCE_MF plays a crucial role in MetS prediction independently of age, sex, and BMI and suggest that IS_SC contributes more significantly than traditional HR-based markers, including RA_HR and L5_HR.

Finally, the comparison between the statistical methods and the XAI-based importance analysis revealed that sleep-related indicators, which did not achieve statistical significance, emerged among the top 10 indicators in the XAI-based analysis. These findings underscore the value of explainable artificial intelligence methods in uncovering hidden patterns that may be missed by traditional statistical approaches.

## Discussion

### Primary Findings

Currently, studies analyzing circadian rhythm digital markers related to MetS are still in their early stages. Previous research has primarily focused on individual factors such as physical activity levels and sleep patterns, but in-depth analysis of circadian rhythm markers for metabolic diseases using wearable devices has rarely been conducted [26-28]. This study aimed to identify and analyze circadian rhythm indicators related to MetS using data from wearable devices.

In addition to existing circadian rhythm indicators, recent studies are proposing new indicators tailored to each target disease area. Table 4 shows studies focused on wearable-based sleep and circadian rhythm analysis. For instance, Cui proposed CARE as a circadian rhythm marker for cognitive function, comparing it with existing indicators like RA [10]. Similarly, Shim introduced a new marker called CosinorAge, which analyzes the relationship between aging and mortality, in addition to Cosinor-based indices such as MESOR, amplitude, and acrophase [12]. In this study, we proposed a time-frequency analysis-based circadian rhythm indicator called CCE and compared it with indicators used in previous studies. As a marker, CARE analyzes circadian rhythms using an energy-based approach, similar to the CCE marker. However, although CARE proved to be a strong marker for cognitive function, it did not show high significance in MetS.

**Table 4.** Previous studies on sleep and circadian rhythm indicator analysis using wrist-worn wearable devices. A summary of studies investigating sleep and circadian rhythm indicators in various health conditions using wearable devices. Each study includes target condition, device type, key indicators analyzed, and methodology used. Indicators include sleep parameters (eg, sleep duration and onset latency), circadian rhythm markers (eg, midline estimating statistic of rhythm, amplitude, and interdaily stability), and novel metrics (eg, circadian activity rhythm energy and midfrequency circadian rhythm energy).

Author	Target	Device	Indicator	Methodology
Kiss et al 2024 [7]	Obesity	Fitbit Charge HR^a^ 2	Sleep onset, sleep offset, sleep duration, sleep onset latency, sleep inertia, MST^b^, total step count, sleeping HR, and resting HR	Explainable boosting machine
Zhang et al 2024 [8]	Major depressive disorder	Fitbit Charge HR 2 or 3	Sleep duration, sleep onset, sleep offset, sleep variability, daily step, step IV, step IS, L5 onset, M10 onset, HR MESOR^c^, HR amplitude, and HR acrophase	Linear mixed-effects models
Ali et al 2023 [9]	Major depressive disorder	Actigraphy ActiCal (Philips)	MESOR, Amplitude, CQ^d^, Acrophase, M10, L5, RA^e^, IV^f^, and IS^g^	Two-tailed *t* test and Mann-Whitney *U* test, and Kolmogorov-Smirnov test
Cui et al 2023 [10]	Cognitive Function	AX3 (Activity)	CARE^h^, and RA	Regression models
Ravindra et al 2023 [11]	Prematurity	Motionwatch8 (CamNTech)	RA, IS, and IV	Deep learning and novel interpretability algorithms
Shim et al 2024 [12]	Aging and Mortality	AX3 (Axivity) or GT3X+ (ActiGraph)	MESOR, amplitude, acrophase, and CosinorAge	Harrel concordance index and Akaike information criterion
Kim et al 2022 [26]	MetS	GalaxyWatch Active1	Walking hours, physical activity hours, and step counts	Paired *t* test and chi-square test
Yamaga et al 2023 [27]	MetS	Fitbit Versa	TST^i^, total step count, and total activity minutes	Multilevel mixed-effects logistic regression
Ours	MetS	Fitbit Versa or Inspire 2	MST, TST, MESOR, amplitude, acrophase, CQ, L5, M10, RA, IS, IV, CARE, and CCE^j^	XAI^k^ models such as SHAP^l^, EBM^m^, and TabNet

a HR: heart rate.

b MST: midsleep time.

c MESOR: midline estimating statistic of rhythm.

d CQ: circadian quotient.

e RA: relative amplitude.

f IV: interdaily variability.

g IS: interdaily stability.

h CARE: circadian activity rhythm energy.

i TST: total sleep time.

j CCE: circadian rhythm energy.

k XAI: explainable artificial intelligence.

l SHAP: Shapley Additive Explanations.

m EBM: explainable boosting machine.

Existing statistical analysis methods may have limitations in detecting complex temporal patterns or interactions. In contrast, XAI offers clearer insights into hidden data patterns by making the predictions of complex models explainable [29]. Kiss et al [7] analyzed the relationship between sleep and HR indicators and obesity through EBM, while Ravindra et al [11] investigated circadian rhythm indicators for prematurity using novel interpretability algorithms that integrate unsupervised clustering, model error analysis, feature attribution, and automated actigraphy analysis. This highlights the growing importance of using XAI to analyze target diseases and circadian markers. Our study aims to address the limitations of previous research by examining the significance of circadian rhythm indicators related to MetS using XAI models such as SHAP, EBM, and TabNet. Analyzing the contribution and significance of circadian rhythm indicators across various models enhances the development of objective markers that more effectively explain MetS.

XAI-based analysis of circadian rhythm indicators revealed that CCE_MF is the most important marker for identifying circadian patterns related to MetS. A decrease in CCE_MF with a 1-hour cycle correlated with an increased contribution to MetS. The HR variability observed in the 1-hour cycle can be related to exercise, physical activity, eating, digestion, and fluctuations in the autonomic nervous system [30,31]. A low energy level in the 1h cycle indicates minimal amplitude or frequency components, suggesting strong physical activity, low metabolic changes, or an absence of exercise. Our study also noted low step counts in the same cycle. Additionally, the RA_HR consistently ranked high across all models and displayed low values in MetS. A low RA_HR indicates reduced HR variability, which can act as a cardiovascular risk factor. For example, HR variability tends to decrease in conditions such as hypertension, MetS, or heart failure [32].

XAI methods, including SHAP, EBM, and TabNet, enabled us to comprehensively understand the contribution of individual indicators to MetS prediction. While SHAP and EBM, both tree-based models, identified similar HR and sleep-related indicators, TabNet, a deep learning approach, highlighted a different set of significant sleep-related indicators. Notably, some sleep-related indicators that did not achieve statistical significance in traditional analyses emerged among the top indicators in the XAI method. This underscores the limitations of conventional statistical approaches that may overlook essential relationships and interactions among variables. The ability of XAI to uncover these hidden patterns suggests its valuable role in advancing research on MetS and related diseases.

Adding CCE markers to existing markers and comparing the results of MetS prediction can be another method to demonstrate the importance of CCE markers. The results of predicting MetS with sleep and circadian rhythm markers showed that incorporating the CCE marker into the EBM model increased the prediction accuracy from 64.31% to 65.44%, while the RF model showed the highest prediction accuracy, increasing from 68.38% to 69.47%. When sex, age, height, weight, and BMI were included, the accuracy increased from 88.23% to 88.93% in the EBM model, and the highest model showed an increase from 89.70% to 90.43% in XGBoost. All prediction results were validated using five-fold cross-validation. In a previous study on obesity prediction, the prediction accuracy of wearable devices and questionnaires (such as age, sex, race, income, and education level) was 72.6% when the EBM model was used. Although new markers have been proposed in various fields, they have not achieved a high level of prediction, and the proposed CCE model showed an increase in prediction accuracy of 1% but did not significantly improve overall performance. However, the introduction of new markers provides valuable insights by explaining MetS through interpreting circadian rhythms, rather than relying simply on existing known factors such as sex, age, and BMI.

The growing prevalence of wearable devices, such as smart bands and watches, is fostering an environment where individuals’ everyday health information can be collected and analyzed in real time. These data could be instrumental in personalized health care, particularly in the early detection and prevention of chronic diseases such as MetS. Keshet provided an overview of wearable and digital devices capable of alerting individuals to specific metabolic outcomes, emphasizing the unique opportunities for creating personalized prevention and treatment strategies for cardiometabolic diseases [2]. This study may contribute to the healthcare landscape by developing wearable data-based circadian rhythm markers, addressing the need for technologies aimed at preventing and treating MetS in everyday life.

### Limitations

This study has some limitations. The dataset used was limited in size and was collected from specific regions or population groups, making the generalization challenging. Further research should delve deeper into investigating the stability and generalizability of the model when considering real-world medical applications. Additionally, there was an issue of data imbalance for MetS and metabolic diseases, potentially leading to model bias toward the majority class. Although ensemble methods such as EBM and XGBoost, which are robust to data imbalance, were used [33], random undersampling was performed five times during model training to further address this issue, and the results were analyzed. In particular, the proposed CCE_MF marker emerged as the most important marker in four of the five tests using SHAP and EBM, and as the second most important marker in one test. However, the most important markers are not consistent in the TabNet model, indicating that a larger dataset is needed for deep learning models to achieve more stable and consistent results. Future studies should further explore deep learning models based on larger datasets.

### Conclusions

The study analyzed a total of 26 digital biomarkers related to MetS using wearable wrist sensor data, with a particular focus on the newly proposed CCE. The XAI techniques, including SHAP, EBM, and TabNet, were applied to assess the significance and importance of each biomarker. Key findings demonstrated that circadian rhythm markers based on HR, particularly CCE and RA_HR, showed high importance across multiple models, reinforcing their potential as robust biomarkers for MetS monitoring. The study also highlighted that traditional sleep markers did not exhibit strong statistical significance, suggesting that circadian rhythm analysis could provide additional insights for MetS research.

## Abbreviations

CARE: circadian activity rhythm energy
CCE: circadian rhythm energy
CCE_MF: midfrequency of CCE
CQ: circadian quotient
CWT: continuous wavelet transform
EBM: explainable boosting machine
GAM: generalized additive model
GLU: Gated Linear Units
HR: heart rate
IS: interdaily stability
IV: intradaily variability
KDCC: The Korean Medicine Daejeon Citizen Cohort
LIME: local interpretable model-agnostic explanation
MESOR: midline estimating statistic of rhythm
MetS: metabolic syndrome
MST: midsleep time
RA: relative amplitude
RA_HR: relative amplitude for heart rate
SHAP: Shapley Additive Explanations
SVD: singular value decomposition
TabNet: Tabular Neural Networks
TST: total sleep time
WT: wavelet transform
XAI: explainable artificial intelligence
XGBoost: extreme gradient boosting
